# Jerusalem artichoke and chungkookjang additively improve insulin secretion and sensitivity in diabetic rats

**DOI:** 10.1186/1743-7075-9-112

**Published:** 2012-12-27

**Authors:** Hye Jeong Yang, Dae Young Kwon, Min Jung Kim, Suna Kang, Da Sol Kim, Sunmin Park

**Affiliations:** 1 Food Functional Research Division, Korean Food Research Institutes, Sungnam, South Korea; 2 Department of Food and Nutrition, Basic Science Institutes, Hoseo University, Asan, South Korea

**Keywords:** Jerusalem artichokes, Chungkookjang, Insulin resistance, Insulin secretion, Islet morphometry

## Abstract

Jerusalem artichoke *(Helianthus tuberosus Linne*, HTL) and chungkookjang (CKJ; fermented soybeans) both modulate energy and glucose metabolism. However, the mechanism and their additive effects are unknown. We investigated whether the consumption of HTL and CKJ altered insulin sensitivity, insulin secretion capacity and β-cell survival in type 2 diabetic animals. Rats were divided into partially pancreatectomized (Px) diabetic rats, and sham operated non-diabetic control rats and all fed high fat diets. Diabetic rats were sub-divided into an untreated diabetic control group and those fed 5% HTL, 5% CKJ or 5% HTL+5% CKJ for 8 weeks. HTL+CKJ treatment reduced visceral fat without modulating energy intake compared to the diabetic-control. Glucose tolerance was improved in an ascending order of diabetic-control, CKJ, HTL, HTL+CKJ, and normal-control, but by different mechanisms. CKJ and CKJ+HTL, but not HTL, increased first and second phase insulin secretion in comparison to the diabetic-control at hyperglycemic clamp. However, glucose infusion rates (mg/kg bw/min) were increased by and CKJ+HTL (13.5), but not HTL (9.4) or CKJ (9.5) alone, and HTL and CKJ+ HTL decreased hepatic glucose compared to diabetic-control during the hyperinsulinemic euglycemic study and were associated with decreased triglyceride accumulation and increased glycogen storage. The improved hepatic insulin sensitivity by HTL and CKJ+HTL was explained by potentiated insulin signaling (tyrosine phosphorylation of insulin receptor substrate 2→phosphorylation of Akt) and phosphorylation of AMPK→phosphorykation of acetyl Co carboxlase in comparison to diabetic-control and decreased PEPCK expression. Absolute β-cell mass was increased by CKJ (23.4mg) and CKJ+HTL (26.3 mg) by increasing proliferation compared to the diabetic-control (21.26 mg). Although HTL lowered β-cell apoptosis, it did not increase β-cell mass (20.8 mg). In conclusions, HTL and CKJ enhanced glucose tolerance in different manners, and exhibited partially additive and complementary effects by reversing insulin resistance and enhancing β-cell function in diabetic rats.

## Introduction

East Asians have traditionally maintained good glycemic control [1]. However, the incidence of diabetes is increasing due to low quality diets early in life and greater consumption of animal fats and less activity in later life [2,3]; changes which exacerbate insulin resistance [3] and ultimately lead to type 2 diabetes [2,4]. Insulin resistant Caucasians develop hyperinsulinemia to maintain glucose intolerance which delays the onset of type 2 diabetes [5]. However, East Asians have lower insulin secretory capacity to compensate for insulin resistance and therefore easily develop type 2 diabetes [4]. Therefore, preventing insulin resistance and potentiating β-cell function and mass is even more important for preventing type 2 diabetes in East Asians, since they have little excess insulin secretory capacity to buffer the effects of insulin resistance.

It is difficult to reverse modern dietary and lifestyle trends, therefore it is important to identify dietary interventions that can prevent or delay the onset of diabetes. We previously reported that 8 week consumption of the fermented soybean food, chungkookjang (20% of the diet), increases β-cell function and mass and attenuates insulin resistance in type 2 diabetic rats [6,7]. However, chungkookjang had only a small effect on insulin sensitivity, so foods that decrease insulin resistance would be a valuable addition to fermented soybeans. Jerusalem artichoke (*Helianthus tuberosus Linne*) is a native plant to North America in the daisy family; its tubers are available in the produce section of many grocery stores in worldwide including Korea and are a common commercial source of inulin. Jerusalem artichoke may improve insulin sensitivity since inulin, its major component, decreases the synthesis of triglycerides and fatty acids in the liver and lowers their circulating levels in rats [8,9]. In addition, it decreases fasting serum glucose levels in healthy humans [10], which may be related to its fructan and coumarins such as ayapin and scopletin [8]. The 20% chungkookjang used in the previous study is more than would be consumed in a typical human diet. Therefore, the anti-diabetic effects of moderate amounts of chungkookjang (soybeans fermented with *Bacilli* spp.) and Jerusalem artichoke were determined in type 2 diabetic rats and some of the potential mechanisms explored.

## Materials and methods

### Chungkookjang and Jerusalem artichokes

Traditionally prepared Chungkookjang was manufactured at Midari Farm (Yeongwol county, Kangwon-Do, Korea). Cooked soybeans were fermented with *Bacillus spp.* at 30°C for 43 h and then freeze-dried and powered for adding into the diet. The process of making CKJ was optimized for anti-diabetic activity based on previous studies [11,12].

Since fructoligossacharides can be easily degraded into free fructose by inulinase after harvesting, the Jerusalem artichokes from Midari Farm (Yeongwol county) were immediately sliced and steamed to inactivate inulinase, and then dried at 60°C and powdered. The Jerusalem artichoke used in this study contained less 4% of free fructose of Jerusalem artichoke powder.

### Isoflavonoids and peptides from chungkookjang and fructoligosaccharides from Jerusalem artichokes

The lyophilized chungkookjang (CKJ) was extracted in 70% methanol containing 0.1% acetic acid; isoflavonoids in the supernatant of the extracts were detected using HPLC (PU 980, JASCO, Japan) equipped with an ODS A303 (4.6 × 250 mm, 5 μm, YMC, USA) reversed phase column and monitored at a wavelength of 254 nm with a UV detector. Elution was carried out at a flow rate of 1.0 ml·min^-1^ with water and acetonitrile containing 0.1% acetic acid. Peaks in each extract were identified by comparing to 12 reference isoflavonoids: genistein, daidzein, and glycitein from Sigma Co. (St. Louis, MO); genistin, daidzin, and glycitin from Indofine (Hillsborough, NJ); and malonyl genistin, malonyl daidzin, malonyl glycitin, acetyl genistin, acetyl daidzin and acetyl glycitin from LC Lab (Woburn, MA).

Peptide contents of chungkookjang were quantified using a ninhydrin method described previously [13] and quantified using an external standard, L-leucine. The profiles of peptides were determined by ultra performance liquid chromatography (UPLC, Waters Co.) using Acquity UPLC BEH C_18_ (2.1 × 100 mm, 1.7 μm; Waters, Milford, MA, USA) and monitored at a wavelength of 220 nm using a PDA detector. Elution was carried out at a flow rate of 0.35 ml·min^-1^ with gradient solution of 0.1% trifluroacetic acid in water and 0.1% trifluroacetic acid in acetonitrile.

Dried Jerusalem artichokes (HTL) were extracted with water at 95°C for 4 h and the extracts were filtered. The filtrates were eluted in an HP-20 column (Supelco) and concentrated with a vacuum evaporator. The concentrates were precipitated with 50% MeOH with inulin in the precipitate and oligosaccharides in the supernatant, which were measured by drying and weighing.

### Animals and diets

Male Sprague Dawley rats, weighing 203±14 g, were housed individually in stainless steel cages in a temperature- and humidity-controlled environment (23°C and 60%) on a normal 12 hour light–dark cycle. All surgical and experimental procedures were performed in accordance with the recommendations found in the Guide for the Care and Use of Laboratory Animals published by the National Institutes of Health, USA, and approved by the Institutional Animal Care and Use Committee of Hoseo University, Korea (2010–08). Mild type 2 diabetes was induced by removing 90% of the pancreas using the Hosokawa technique [14]. After 1 week of recovery, the pancreatectomized (Px) rats were excluded from the study if their random-fed serum glucose levels were less than 7 mmol/L; the rats included in the study had serum glucose levels of 9.4-11.8 mmol/L without fasting. During the recovery period, the pancreas of Px rats regenerate to about 50% of the original mass, and their insulin secretory capacity was about 40-50% lower than sham-operated (Sham) non-diabetic rats that had the same operation as Px rats, except without removing the pancreas. Px rats did not develop any symptoms associated with nutrient malabsorption or ketosis and the ratio of α- and β-cells was similar to other type 2 diabetic animals. Therefore, Px rats exhibited a similar phenotype to Asian type 2 diabetes characterized by normal adiposity, insulin deficiency and insulin resistance [14-16].

The Px rats were randomly assigned to four different groups (diabetic-control, CKJ, CKJ+HTL, and HTL) of 20 animals. The diabetic-control and non-diabetic Sham (normal-control) groups had a 40% energy high-fat diet without chungkookjang and Jerusalem artichoke. All Px and Sham rats freely consumed water and corresponding modified AIN-93 semi-purified diets for 8 wk [17]. CKJ, CKJ+HTL and HTL diets contained 5% lyophilized chungkookjang, 5% lyophilized chungkookjang+5% lyophilized Jerusalem artichoke, or 5% lyophilized Jerusalem artichoke, respectively. Since CKJ and HTL contained a mixture of carbohydrates, protein, and lipids, their compositions were analyzed and the macronutrient compositions adjusted to equal proportions by adding soybean oil and cellulose. All diets consisted of approximately 42 energy percent (En%) carbohydrates, 18 En% protein, and 40 En% fats (Table 1) in order to study the effect of CKJ and HTL on insulin sensitizing and insulinotropic actions under an aggravated diabetic condition. The degree of hydrolysis of protein concentration, types of isoflavones and dietary fiber were the main differences among diets. Isoflavonoid contents were measured in our previous study; total isoflavonoids were decreased but isoflavonoid aglycones increased during fermentation (Table 1). 

**Table 1:** Composition of experimental diets

	**Casein diet**	**Chungkookjang (CKJ) diet**	**Jerusalem artichoke (HTL) diet**	**CKJ+HTL diet**
Casein (g)	200	183	197	183
Methionine (g)	3	3	3	3
Corn starch (g)	300	295	273	260
Sucrose (g)	200	200	200	200
Cellulose (g)	34	14	14	0
Corn oil (g)	50	44	50	43
Shortening (g)	150	150	150	150
Mineral (g)	35	33	35	33
Vitamin (g)	10	10	10	10
Choline (g)	2	2	2	2
Powder of CKJ (g)	0	50	0	50
Powder of HTL (g)	0	0	50	50
Total isoflavonoids (%)	-	0.013	-	0.013
Isoflavonoid aglycones (%)	-	0.012	-	0.012
Fructooligossaccharides including inulin (%)			1.5	1.5

After 16 h overnight-fasting, serum glucose levels and body weights of all experimental animals were measured every Tuesday at 10 AM. To avoid fasting effects on determining food intake, weighed feed was provided each day and remaining food weighed the next, daily feed intake was the average over one week. An oral glucose tolerance test (OGTT) was performed every three weeks in overnight-fasted animals by orally administering 2 g glucose/kg body weight. Serum glucose and insulin were measured by tail bleeding at 0, 10, 20, 30, 45, 60, 90 and 120 min after glucose loading, and the average of the area under the curve of serum glucose and insulin was calculated. Serum glucose levels were analyzed with a Glucose Analyzer II (Beckman, Palo Alto, CA), and serum insulin and leptin levels were measured by radioimmunoassay kit (Linco Research, Billerica, MA).

### Hyperglycemic clamp

After seven weeks of treatment, catheters were surgically implanted into the right carotid artery and left jugular vein of 10 rats from each group after anesthetization (ketamine 100 mg/kg bw and xylazine 10 mg/kg bw for all anesthesia). After 5–6 days of implantation, a hyperglycemic clamp was performed after 16 h fasting to determine insulin secretory capacity as previously described [16,18]. During the clamp, 25% glucose solution was infused to maintain serum glucose levels of 5.5 mM above baseline by measuring Glucose Analyzer II (Beckman) at 0, 2, 5, 10, 60, 90 and 120 mins and at those times serum insulin levels were measured by radioimmunoassay (Linco Research). After the clamp, rats were freely provided with feed and water for 2 days and the next day, they were fasted 16 hours, anesthetized, and human regular insulin (5 U/kg body weight) was injected through the inferior vena cava. Ten min later, they were killed by decapitation and tissues were rapidly collected, frozen in liquid nitrogen, and stored at −70°C for further experiments.

### Hyperinsulinemic euglycemic clamp

After catheterization on week 7, a hyperinsulinemic euglycemic clamp was performed on 16 h-fasted conscious rats from each group to determine insulin resistance as previously described [19]. [3-^3^H]glucose (NEN Life Science, Boston, MA) was continuously infused during a 4-hour period at the rate of 0.05 μCi·min^-1^. Basal hepatic glucose output was measured in blood collected at 100 and 120 minutes after initiation of the [3-^3^H] glucose infusion. Then a primed continuous infusion of human regular insulin (Humulin; Eli Lilly, Indianapolis, IN) was initiated at a rate of 20 pmol·kg^–1^·min^–1^ to raise plasma insulin concentration to approximately 1100 pM at 210 – 240 min. While infusing exogenous insulin and 25% glucose solution, blood samples from arteries were collected at 10-minute intervals for glucose estimation by Glucose Analyzer II (Beckman). Glucose solution was infused at variable rates as needed to clamp euglycemia at approximately 6 mmol/L. For the determination of plasma [3-^3^H]glucose concentrations, plasma was deproteinized with ZnSO_4_ and Ba(OH)_2_, dried to remove ^3^H_2_O, re-suspended in water, and disintegrations per min (dpm) of ^3^H were recorded. The plasma concentration of ^3^H_2_O was determined by the difference between ^3^H counts with and without drying. Rates of whole body glucose uptake and basal glucose turnover were determined as the ratio of the ^3^H] glucose infusion rate to the specific activity of plasma glucose (dpm/μmol) during the final 30 minutes of the respective experiments. Hepatic glucose production at hyperinsulinemic clamped state was determined by subtracting the glucose infusion rate from the whole body glucose uptake.

### Islet morphometry

Six rats from each group were treated with 5-bromo-2-deoxyuridine (BrdU; Roche Molecular Biochemicals, Indianapolis, Indiana, USA; 100 μg/kg body weight) at the end of the 12-week experimental period. Six hours post-injection, pancreas samples were prepared and analyzed as previously described [16]. Each pancreas was dissected, fixed in a 4% paraformaldehyde solution (pH 7.2) overnight at room temperature, and embedded in paraffin blocks. To prevent twice selecting sections (5-μm) with the same islet, after rehydration every sixth or seventh section was selected to determine β-cell area, BrdU incorporation and cell death. Endocrine β-cells were identified by applying a guinea pig anti-insulin antibody in paraffin-embedded pancreatic sections by immunohistochemistry. Beta-cell areas were measured by acquiring images from eight to ten distal, random, non-overlapping images at × 20 of insulin stained pancreatic sections and the area was measured by densitometery. Results of β-cell quantification are expressed as the percentage of the total surveyed pancreas area containing insulin-positive cells. The β-cell mass was measured by multiplying average β-cell area by pancreas weight. The α-cells were determined by immunostaining with a rabbit anti-glucagon in paraffin-embedded pancreatic section. The ratio of β- and α-cells was calculated. Beta-cell proliferation was examined by the incorporation of BrdU as determined by performing a double-label immunohistochemistry with anti-insulin (Zymed Laboratories, South San Francisco, CA) and anti-BrdU antibodies (Roche, Mannheim, Germany) on rehydrated paraffin sections. Cell death of β-cells was measured by TUNEL kit (Roche) in paraffin sections of the pancreas and counterstained with hematoxylin and eosin to visualize islets [20].

### Immunoblot analysis

Livers collected from rats stimulated with insulin for 10 min were lysed in 20 mM Tris buffer (pH 7.4) containing 2 mM EGTA, 137 mM NaCl, 1% NP40, 10% glycerol, and 12 mM α-glycerol phosphate and protease inhibitors. Immunoblotting was previously described [7]. Glycogen contents in the livers were determined by centrifuging liver lysates at 4000 × g for 10 minutes after which supernatants were deproteinized with 1.5 N perchloric acid. The glycogen content was calculated from glucose concentrations derived from glycogen hydrolyzed by α-amyloglucosidase in an acid buffer [21]. Triacylglycerol was extracted with chloroform-methanol (2:1, vol/vol) from the liver and resuspended in pure chloroform [22]. After evaporating chloroform, the residue was suspended with PBS and 0.1% triton X-100 and the suspension was sonicated and boiled for 5 min. The triacylglycerol contents of the suspensions were assayed using a Trinder kit (Young Dong Pharm., Seoul, Korea).

### Statistical analysis

Statistical analysis was performed using SAS software and all results are expressed as mean±SD. Anti-diabetic effects of Px-control, CKJ, HTL, CKJ+HTL and normal-control were determined by one-way analysis of variance. Significant differences in the main effects among groups were identified by Tukey tests. P<0.05 was considered statistically significant.

## Results

### Isoflavonoids and fructan contents in HTL and CKJ

CKJ contained both isoflavonoid glycosides and aglycones, with similar diadzein and daidzin concentrations but much higher concentrations of genistein than genistin (Table 2). HTL contained inulin and fructoligossacharides (Table 2).

**Table 2:** Isoflavonoids, peptides and fructoligossacharides (mg/g dry matter)

	**Chungkookjang diet**	**Jerusalem artichoke diet**
Daidzin	0.105±0.02	-
Genistin	0.014±0.002	-
Glycitin	0.119±0.02	-
Total isoflavonoid glycosides	0.458±0.007	-
Daidzein	0.111±0.02	-
Genistein	0.082±0.01	-
Glycitein	0.056±0.005	-
Total isoflavonoid aglycones	0.249±0.03	-
Peptides	109.6±5.96	-
Inulin	-	75
Fructoligosccharides	-	225

### HTL and CKJ lowers body weight and fasted glucose concentrations

Body weight was higher in the rats of the normal-control group than those of the diabetic groups despite lower energy intake but it was not significantly different among the diabetic groups (Table 3). Unlike body weight, epididymal fat pads and serum leptin levels decreased in a descending order of normal-control > diabetic-control > CKJ, HTL > CKJ+ HTL, although for epididymal fat pads the only significant difference among diabetic rats was the lower CKJ+HTL weights than diabetic-controls. Serum triglyceride levels were higher in the diabetic-control group than the normal-control group while they were lowered with HTL and CKJ and the mixture of CKJ and HTL did not have an additive effect on serum triglyceride levels. As expected, rats in the normal-control had the lowest fasting serum glucose levels while CKJ and HTL also had lower levels than the diabetic-control and was lower in CKJ+HTL than either alone. The overnight-fasted serum insulin levels were higher in CKJ, but not HTL, compared to the diabetic-control and rats in the CKJ+HTL group had higher levels than those fed CKJ alone (Table 3). Thus, CKJ and HTL lowered serum glucose levels in different manners. The overnight-fasted serum glucagon levels of diabetic rats were not significantly different from those of non-diabetic rats and they were also not affected by CKJ or HTL (Table 3).

**Table 3:** Metabolic parameters after overnight-fasting

	**Diabetic-CON (n=20)**	**CKJ (n=20)**	**HTL (n=20)**	**CKJ+HTL (n=20)**	**Normal-CON (n=20)**
Body weight (g)	337±47^b^	320±38^b^	324±33^b^	317±39^b^	427±39^a*^
Epidydimal fat pads (g)	3.0±0.5^b^	2.7±0.4^bc^	2.7±0.4^bc^	2.4±0.4^c^	4.6±0.7^a*^
Caloric intakes (Kcal/day)	115±18^b^	109±17^b^	110±16^b^	105±17^b^	93±14^a*^
Serum triglyceride levels (mmol/L)	1.26±0.23^a^	1.01±0.12^b^	0.98±0.14^b^	0.97±0.15^b^	0.93±0.13^b*^
Serum leptin levels (ng/mL)	5.1±0.7^b^	4.3±0.6^c^	4.4±0.6^c^	3.6±0.6^d^	6.6±0.8^a*^
Serum glucose levels (mmol/L)	9.8±1.4^a^	8.1±1.0^b^	7.7±0.9^b^	6.3±0.8^c^	4.3±0.7^d*^
Serum insulin levels (ng/mL)	0.69±0.13^d^	0.81±0.13^c^	0.66±0.11^d^	1.02±0.16^b^	1.38±0.24^a*^
Serum glucagon (ng/mL)	95.4±10.3	92.5±11.7	91.7±12.1	91.5±10.9	90.4±11.5

Values are mean±SD. Rats in the normal-control (Normal-CON) and diabetic-control (Diabetic-CON) was fed high fat diets while diabetic rats in the CKJ, HTL, and CKJ+HTL groups were provided with high fat diets supplemented with 5% CKJ, 5% HTL, and 5% CKJ+ 5% HTL, respectively.

^*^Significantly different among the groups at p<0.05 by one-way ANOVA.

^a,b,c,d^Values in the same row with different superscripts were significantly different in Tukey test at P<0.05.

### CKJ, HTL and CKJ+HTL improve glucose tolerance in different manners

**Figure 1:**
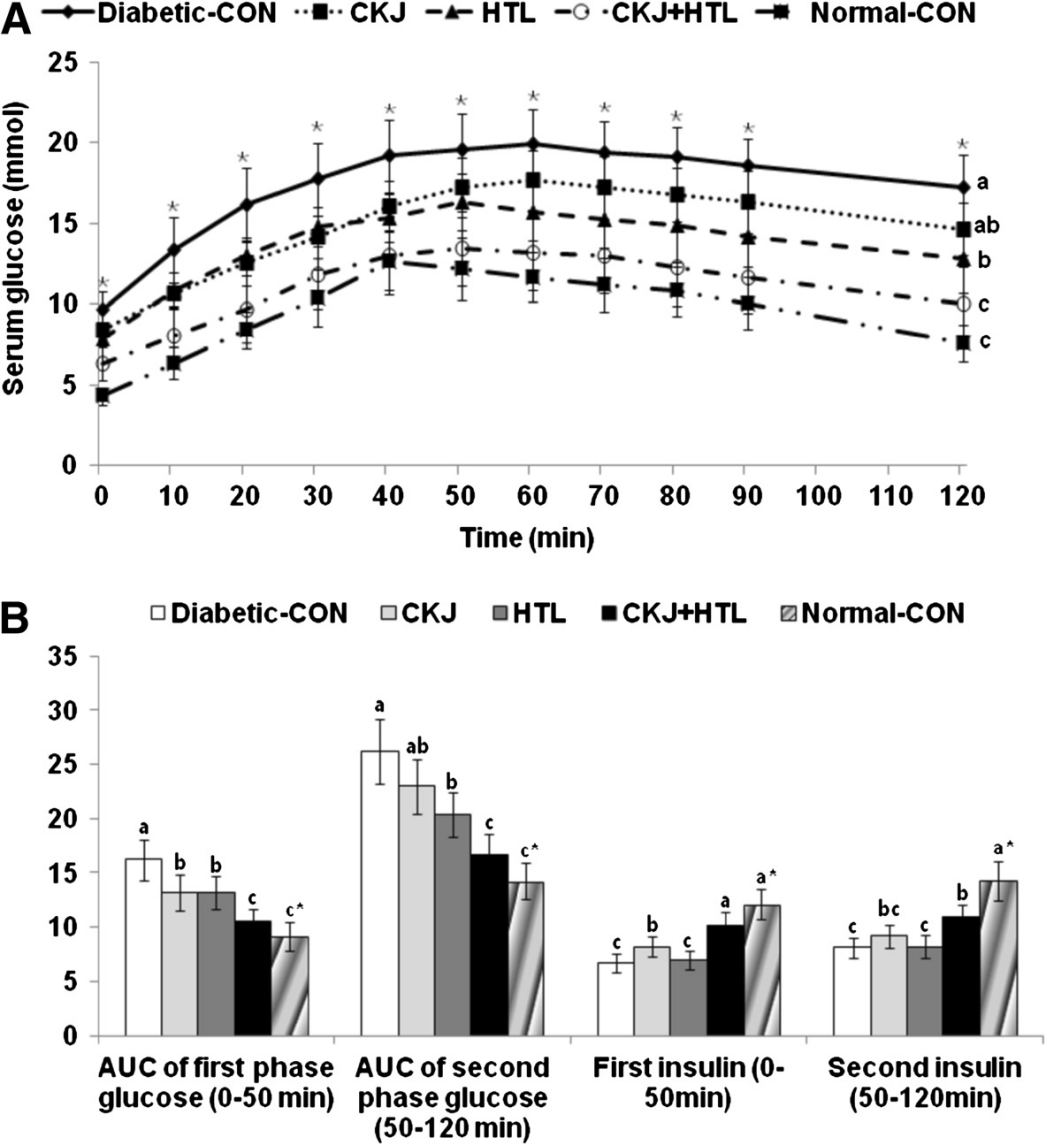
**Serum glucose levels (A) and the area under the curve of serum glucose and insulin (B) during oral glucose tolerance tests.** Serum glucose time course (A) and area under the curve (AUC) for glucose and insulin in non-diabetic Sham and diabetic Px rats fed high fat diets with casein (normal-control and diabetic-control groups, respectively), 5% CKJ, 5% HTL, or 5% CKJ+ 5% HTL for 8 wk, following oral loading with 2 g glucose per kg bw. Normal-CON group represented non-diabetic Sham rats fed high fat diets with casein. The sample size was the same as in Table 3. The bars represent mean±SD. ^*^Significantly different among the groups at p<0.05 by one-way ANOVA. ^a,b,c^Values in the same row with different superscripts were significantly different in Tukey test at P<0.05.

Rats in the diabetic-control had much higher peak serum glucose levels which decreased slowly compared to those in the normal-control (Figure 1A). In CKJ and HTL and especially CKJ+HTL rats, peak serum glucose levels were lower and decreased faster than controls (Figure 1A). Diabetic-control rats exhibited much lower serum insulin levels in first and second parts of OGTT than normal-control rats (Figure 1B). Area under the curve (AUC) of insulin in the first part was higher in ascending order of the diabetic-control < CKJ < HTL < CKJ+HTL < normal-control whereas in CKJ+HTL and HTL it decreased in the second part, but not with CKJ alone and normal-control. As the changes in serum glucose levels were associated with insulin secretion and insulin action, only CKJ increased serum insulin levels in both first and second parts of OGTT (Figure 1B), indicating that CKJ and HTL improved glucose tolerance differently.

### CKJ potentiates insulin secretion capacity during hyperglycemic clamp

**Figure 2:**
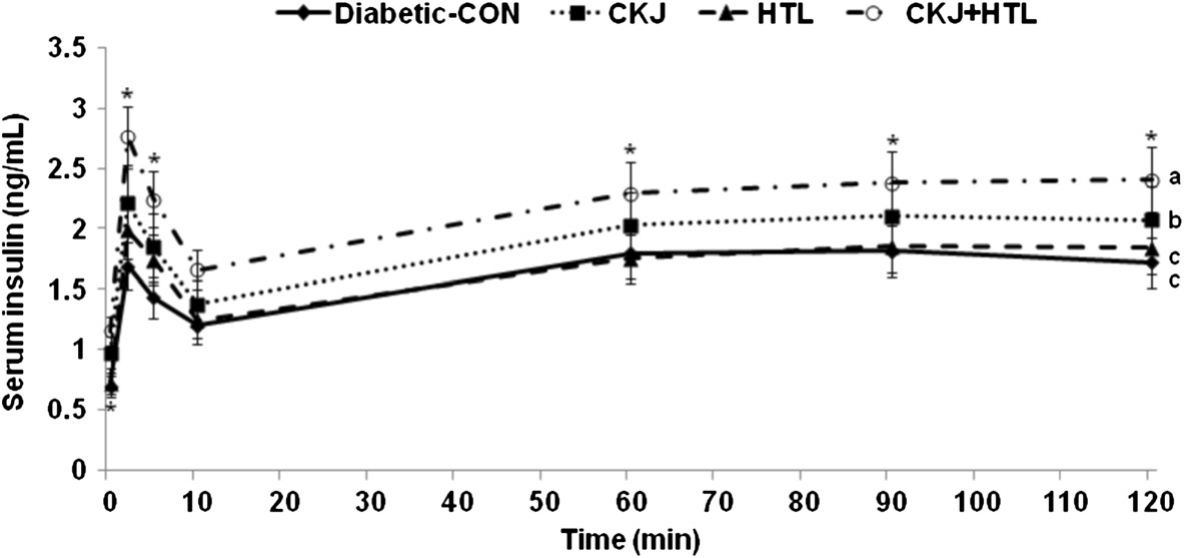
**Insulin secretion capacity during hyperglycemic clamp.** Serum insulin levels were measured during hyperglycemic clamp in diabetic Px rats fed high fat diets with casein (diabetic-CON group), 5% CKJ, 5% HTL, or 5% CKJ+ 5% HTL for 8 wk. Normal-CON group represented non-diabetic Sham rats fed high fat diets with casein. The sample size was the same as in Table 4. ^*^Significantly different among the groups at p<0.05 by one-way ANOVA. ^a,b,c^Values in the same row with different superscripts were significantly different by Tukey test at P<0.05.

In comparison to those in the normal-control group, rats in the diabetic-control group had lower first and second phase insulin secretion by 60.4% and 51.9%, respectively, during hyperglycemic clamp. CKJ and HTL increased first phase insulin secretion more than the diabetic-control and CKJ+HTL elevated it more than CKJ or HTL alone (Figure 2 & Table 4). However, CKJ and HTL+CKJ, not HTL alone, increased second phase insulin secretion and AUC of insulin during hyperglycemic clamp in comparison to the diabetic-control. Glucose infusion rates required to maintain serum glucose levels above 5.5 mM were decreased in a descending order of normal-control > CKJ+HTL > CKJ, HTL and diabetic-control. In addition, insulin sensitivity during hyperglycemic state exhibited the same trend of glucose infusion rates but insulin sensitivity of rats in the HTL group was higher than that in the diabetic-control group and CKJ+HTL was improved the sensitivity as much as the normal-control (Table 4).

**Table 4:** Insulin secretion capacity during hyperglycemic clamp

	**Diabetic-CON (n=10)**	**CKJ (n=10)**	**HTL (n=10)**	**CKJ+HTL (n=10)**	**Normal-CON (n=10)**
Serum insulin at basal state	0.72±0.13^d^	0.84±0.11^c^	0.69±0.10^d^	1.07±0.16^b^	1.36±0.26^a*^
Serum insulin at first phase (ng/mL)	1.56±0.23^d^	2.03±0.28^c^	1.86±0.31^c^	2.50±0.38^b^	3.94±0.65^a*^
Serum insulin at second phase (ng/mL)	1.81±0.31^c^	2.16±0.35^b^	1.80±0.28^c^	2.33±041^b^	3.76±0.67^a*^
Area under the curve of insulin (AU)	195.8±25.4^d^	235.3±34.2^c^	200.0±28.5^d^	266.2±32.5^b^	406.4±65.9^a*^
Glucose infusion rate (mg/kg bw/min)	8.3±1.2^d^	9.5±1.2^c^	9.4±1.2^c^	13.5±1.5^b^	25.5±3.4^a*^
Insulin sensitivity (μmol glucose · min^-1^ · 100 g^-1^ per μmol insulin/L)	12.4±1.5^c^	11.9±1.7^c^	14.1±1.7^bc^	15.6±1.8^b^	18.3±2.6^a*^

Values are mean±SD. First phase insulin secretion was defined as the average of serum insulin levels at 2 and 5 mins, with second phase at 60, 90 and 120 mins. Insulin sensitivity at hyperglycemic state was calculated as the ratio of glucose infusion rates to steady-state serum insulin levels. Rats in the normal-control (Normal-CON) and diabetic-control (Diabetic-CON) was fed high fat diets while diabetic rats in the CKJ, HTL, and CKJ+HTL groups were provided with high fat diets supplemented with 5% CKJ, 5% HTL, and 5% CKJ+ 5% HTL, respectively.

^*^Significantly different among the groups at p<0.05 by one-way ANOVA.

^a,b,c,d^Values in the same row with different superscripts were significantly different in Tukey test at P<0.05.

### CKJ increases β-cell mass

**Figure 3:**
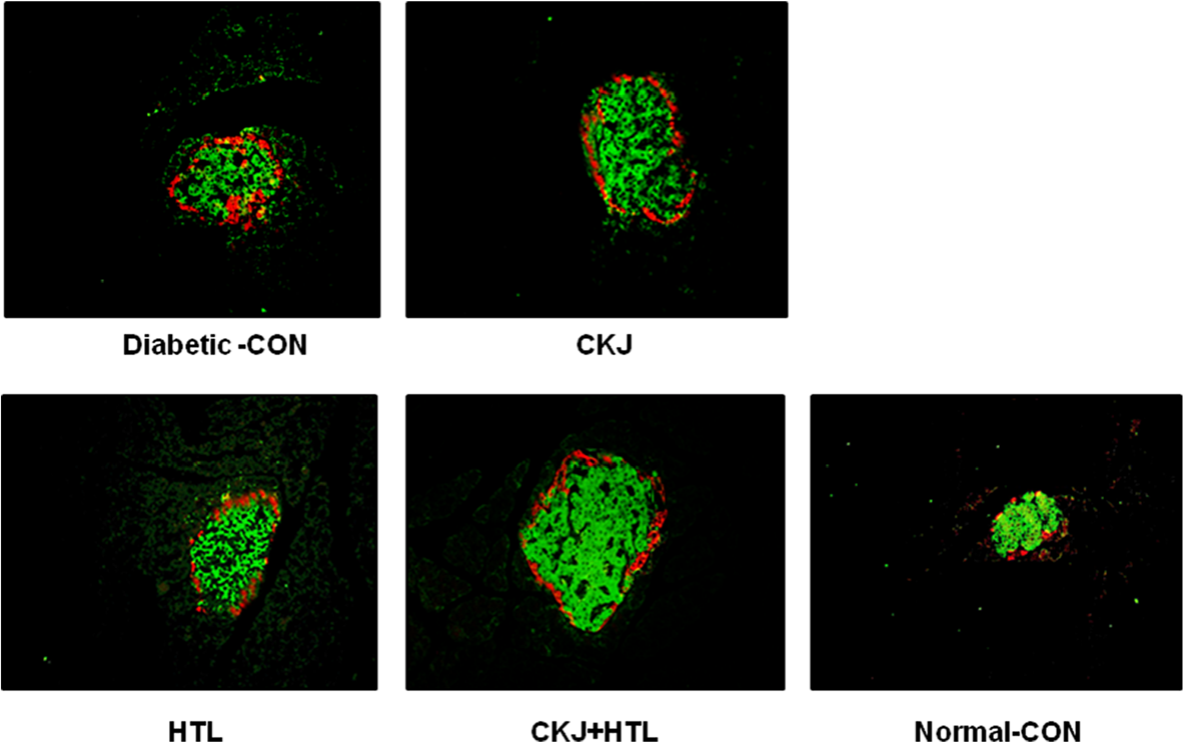
**Islet morphometry representing β-cells and α-cells.** At the end of experiment, the α-cells were determined by immunostaining with a rabbit anti-glucagon in paraffin-embedded pancreatic section. Green and red were immunostained with anti-insulin and anti-glucagon, respectively and they are represented as β-cells and α-cells, respectively.

The β-cell areas were increased in order of normal-control, diabetic-control, HTL, CKJ and CKJ+HTL since diabetic rats needed more β-cells for regenerating the pancreas (Table 5). When calculating β-cell mass by multiplying β-cell area by pancreas weight, β-cell mass was greatest in the normal-control group since pancreas weights of Px rats were about 40-50% of the original (data not shown). The β-cell mass of rats in the diabetic-control group was 64±7.6% less than in the normal-control group. The changes in β-cell area in Px rats was related to increased β-cell proliferation in comparison to Sham rats (Table 5). The normal-control group exhibited less β-cell proliferation than the diabetic-control group while CKJ and CKJ+HTL exhibited increased β-cell proliferation in comparison to the diabetic-control group. However, normal-control, HTL and CKJ+HTL had decreased β-cell apoptosis in comparison to the diabetic-control. The ratio of β:α cells was lower in the diabetic-control group than the normal-control group, indicating that pancreatectomy induced α-cell infiltration into β-cells in islets (Table 5 and Figure 3). CKJ, HTL and CKJ+HTL elevated the ratio of β:α cells compared to the diabetic-control. This indicated that in diabetic rats, CKJ+HTL attenuated α-cell infiltration and improved islet morphometry to resemble normal-control.

**Table 5:** The modulation of islet morphometry

	**Diabetic-CON (n=7)**	**CKJ (n=7)**	**HTL (n=7)**	**CKJ+HTL (n=7)**	**Normal-CON (n=7)**
β-cell area (%)	6.4±0.7^b^	7.0±0.7^ab^	6.6±0.7^b^	7.8±0.8^a^	5.6±0.7^c*^
Individual β-cell size (μm^2^)	242.4±31.1^a^	221.3±25.4^ab^	215.6±24.7^b^	190.5±25.4^b^	182.2±26.8^b*^
Absolute β-cell mass (mg)	21.1±2.6^c^	23.4±2.7^bc^	22.8±2.5^c^	26.3±2.8^b^	34.6±4.8^a*^
BrdU^+^ cells (% BrdU^+^ cells of islets)	0.83±0.10^c^	0.95±0.11^b^	0.86±0.12^bc^	1.15±0.13^a^	0.74±0.09^c*^
Apoptosis (% apoptotic bodies of islets)	0.73±0.08^a^	0.68±0.08^ab^	0.65±0.08^b^	0.60±0.07^b^	0.65±0.10^b*^
Ratio of β:α cells	4.6±0.5^b^	5.8±0.6^b^	5.6±0.6^b^	6.3±0.7^a^	6.0±0.8^a*^

Values are mean±SD. Rats in the normal-control (Normal-CON) and diabetic-control (Diabetic-CON) was fed high fat diets while diabetic rats in the CKJ, HTL, and CKJ+HTL groups were provided with high fat diets supplemented with 5% CKJ, 5% HTL, and 5% CKJ+ 5% HTL, respectively.

^*^Significantly different among the groups at p<0.05 by one-way ANOVA.

^a,b,c^Values in the same row with different superscripts were significantly different in Tukey test at P<0.05.

### HTL attenuates insulin resistance

**Figure 4:**
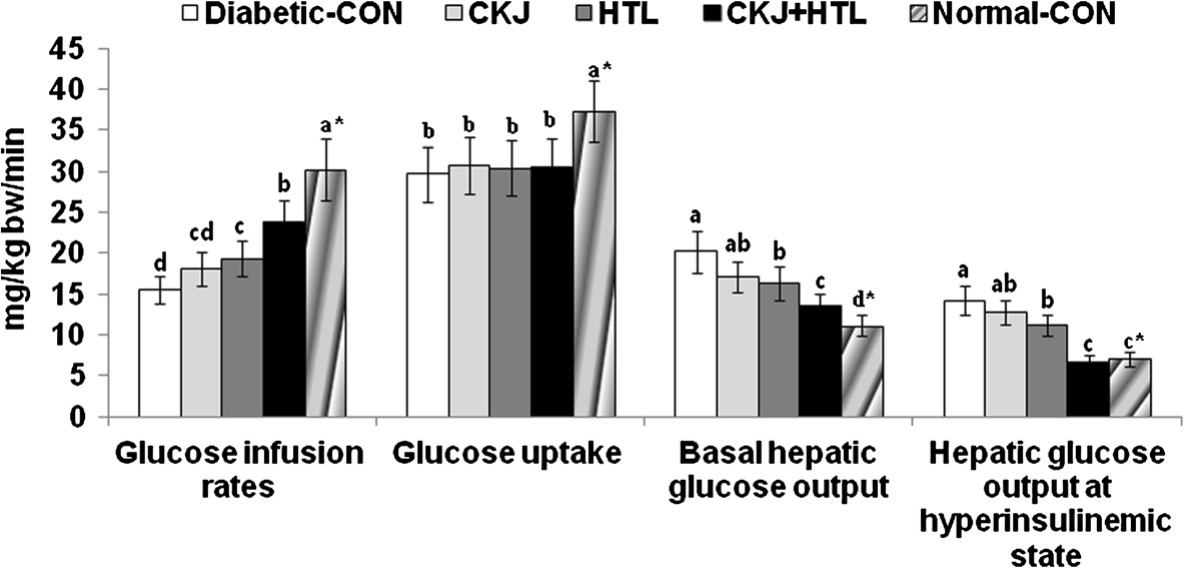
**Metabolic parameters during a euglycemic hyperinsulinemic clamp.** Euglycemic hyperinsulinemic clamp was performed in conscious, free moving and overnight fasted diabetic rats fed high fat diets supplemented with casein (diabetic-CON), 5% CKJ, 5% HTL, and 5% CKJ+5% HTL, for 8 wks to determine whole body insulin resistance. Normal-CON group represented non-diabetic Sham rats fed high fat diets with casein. Glucose infusion rates (GIR), whole body glucose uptake, and hepatic glucose output at basal and clamped states were determined. The sample size was the same as in Table 4. ^*^Significantly different among the all groups at p<0.05 by one-way ANOVA. ^a,b,c,d^Values in the same row with different superscripts were significantly different in Tukey test at P<0.05.

During hyperinsulinemic euglycemic clamp, glucose infusion rates and whole body glucose uptake at about 1100 pM serum insulin levels were lower in rats in the diabetic-control group than the normal-control group (Figure 4). Hyperinsulinemic euglycemic clamp was performed to determine whether the improved glucose tolerance of CKJ, HTL and CKJ+HTL administered rats was associated with ameliorated insulin resistance. The different diets did not alter whole body glucose uptake among the diabetic groups but HTL and CKJ+HTL significantly increased glucose infusion rates (Figure 4). Taken together the glucose infusion rates and whole body glucose uptake, hepatic glucose output at hyperinsulinemic state was lowered in a descending order of the diabetic-control, CKJ > HTL > CKJ+HTL > normal-control (Figure 4).

### CKJ and HTL improve hepatic insulin signaling

**Figure 5:**
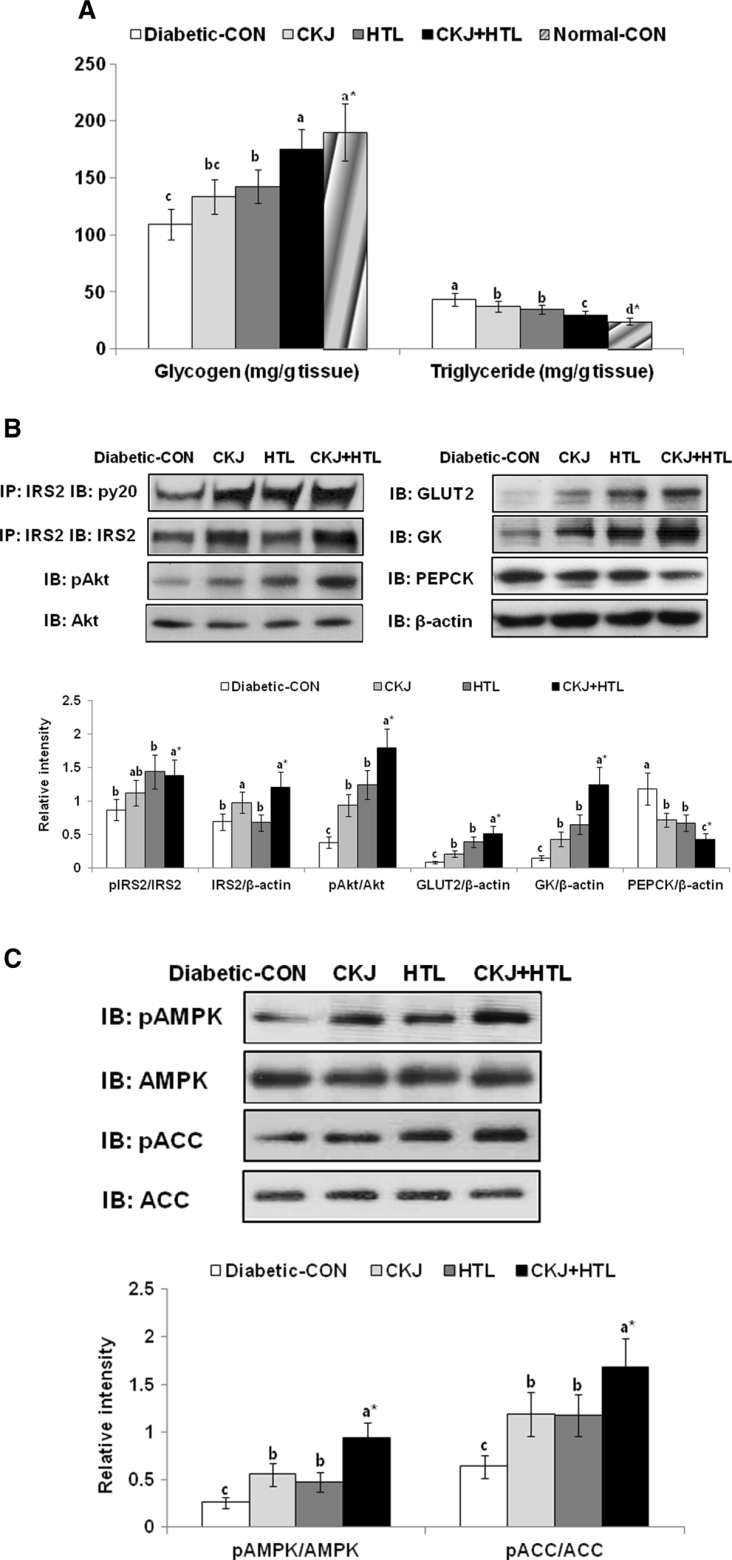
**Glycogen and triglyceride accumulation and insulin signaling in the liver at the end of experiment.** After 8 wk of feeding diabetic rats from each group fed high fat diets with casein (diabetic-CON), 5% CKJ, 5% HTL, or 5% CKJ+5% HTL, the liver was collected after injecting with insulin (5 U/kg body weight) for 10 min and lyzed with designated buffer. Hepatic glycogen and triglyceride contents (**A**), hepatic insulin signaling (**B**) and AMPK signaling (**C**) were determined. Normal-CON group represented non-diabetic Sham rats fed with the casein diet. PEPCK, phosphoenolpyruvate carboxykinase. ^*^Significantly different among the all groups at p<0.05 by one-way ANOVA. ^a,b,c,d^Values in the same row with different superscripts were significantly different in Tukey test at P<0.05.

Glycogen accumulation was higher in the normal-control group than the diabetic-control group (Figure 5A), whereas in diabetic rats HTL and CKJ+HTL increased glycogen storage in the liver, in comparison to the diabetic-control group. Triglyceride storage deceased in descending order of diabetic-control > CKJ, HTL > CKJ+HTL > normal-control. Thus, CKJ+HTL partially normalized hepatic storage of glycogen and triglyceride, approaching that of the normal-control group (Figure 5A).

β-Actin normalized tyrosine phosphorylation of insulin receptor substrate-2 (IRS2), which is the first protein to transmit the insulin receptor signal, was increased with CKJ, HTL, and CKJ+HTL. However, the tyrosine phosphorylation of IRS2 based on IRS2 expression was higher in HTL and CKJ+HTL than the control, but it was not changed with CKJ since IRS2 expression exhibited an increase with CKJ and CKJ+HTL (Figure 5B). Consistent with tyrosine phosphorylation of IRS2, the phosphorylation of Akt, a downstream effector of insulin receptor signaling, was potentiated in the ascending order of diabetic-control, CKJ, HTL, and CKJ+HTL, indicating that CKJ+HTL improved hepatic insulin signaling the most (Figure 5B). The expression of phosphoenolpyruvate carboxykinase (PEPCK), the key regulator of gluconeogenesis, was decreased in the descending order of diabetic-control, CKJ, HTL, and CKJ+HTL (Figure 5B). The expressions of glucose transporter-2 (GLUT2) and glucokinase were also increased in CKJ, HTL and CKJ+HTL in comparison to the diabetic-control (Figure 5B).

AMPK phosphorylation, an intracellular energy gauge, exhibited an ascending order of increase in control < CKJ=HTL < CKJ+HTL (Figure 5C). In addition, the phosphorylation of acetyl CoA carboxylase (ACC), a mediator of AMPK downstream, was consistent with AMPK.

## Discussion

Jerusalem artichoke is high in fructoligosacharides whereas chungkookjang is rich in isoflavonoid aglycones and small peptides. Large dosages of chungkookjang reportedly exert anti-diabetic effects by potentiating glucose-stimulated insulin secretion and attenuating insulin resistance [5,7]. In addition, Jerusalem artichoke had anti-diabetic effects due to high in fructoligossacharides that may decrease insulin resistance by mechanisms that remain unknown. Since chungkookjang and Jerusalem artichoke may have different anti-diabetic mechanisms it is worth evaluating their combined effects. In the present study, HTL and CKJ improved glucose tolerance differently in diabetic rats, and when combined they remarkably enhanced glucose homeostasis in Px rats, type 2 diabetic rats. After removing 90% of the pancreas, the pancreas is regenerated up to 40-50% of the intact pancreas and insulin secretion capacity is about 50-60% of the non-diabetic rats. Thus, Px rats are non-obese and moderate type 2 diabetic animal model. Px rats exhibited a similar phenotype to Asian type 2 diabetes, which has specific characteristics of non-obesity and high susceptibility to insulin deficiency with increased insulin resistance [14-16]. Rather than becoming hyperglycemic, Asians with insulin resistance exhibit normal insulin levels or hypoinsulinemia, and easily progress from glucose intolerance to type 2 diabetes [16]. Px rats are a good model to study β-cell expansion and the relationship between β-cell function and insulin resistance since they are a non-obese type 2 diabetic model with characteristics relevant to Asian type 2 diabetes.

HTL contains fructan which is largely comprised of inulin and oligofructose. Fructan is a non-digestible fiber with health benefits [23-25]. It selectively stimulates the growth and/or activity of some beneficial bacteria in the colon and represses the growth of pathogens [8]. However, the effects of inulin and oligofructose on glucose metabolism are not fully understood and the available data are contradictory. Recently, Dewulf et al. [26,27] revealed that inulin-type diets counteract high-fat diet-induced obesity via suppressing G protein-coupled receptor-43 overexpression through the modification of the gut microbiota and they resulted in a decreased level of circulating lipopolysaccharide and lower C-reactive protein levels to attenuate inflammation. Therefore, the prebiotics such as oligofractan in HTL might reduce systemic inflammation which could be protective against diabetes. Since CKJ contains beneficial *Bacillus* that can use oligofructan in HTL as an energy source, the combination of CKJ and HTL may have a synergistic effect. Some studies have found that in diabetic rats, 10-20% oligofructose diets decrease postprandial glycemia, but the results for serum insulin levels were inconsistent [8]. In healthy humans, 20 g/d oligofructan does not alter fasting serum glucose levels, but in diabetics, 8 g/d oligofructan lowers glucose levels [10,24]. However, Aslan et al. [25] found that 80% ethanol extracts (500 mg/kg body weight) did not improve blood glucose levels after glucose load, perhaps due to higher contents of free fructose resulting from improper storage conditions [28,29]. Improperly stored HTL can break down into simple sugars and exacerbate diabetic symptoms. We found that 5% HTL extracts marginally improved glucose metabolism (data not shown).

Besides lowering postprandial glycemia, oligofructan reduces hepatic gluconeogenesis in normal subjects [10], which may be mediated by short-chain carboxylic acids, especially propionates, made by intestinal microbes from inulin. Boillot et al. [9] reported that propionate consumption reduced fasting blood glucose levels in rats. In addition, propionates inhibit gluconogensesis in isolated hepatocytes when converted into methyl malonyl CoA and succinyl CoA which inhibit pyruvate carboxylase activity. In consistent with other study about oligofructans [10], the present study revealed that HTL improved hepatic insulin sensitivity by enhancing insulin signaling in the liver.

Oligofuctose was shown to increase serum glucose dependent insulinotropic peptides and glucagon-like peptides (GLP-1), regulating post-prandial insulin release and potentiating insulin action by two-fold in rats [30]. However, it is controversial. Parnell and Reimer [31] reported that oligofructan supplementation (21 g/day) for 12 weeks decreased body weight by lowering food intake and serum glucose levels in obese adults but serum GLP-1 levels were not altered. The increased serum GLP-1 levels by oligofructans might be related to the tropic action of short-chain fatty acids produced by colonic oligofructan fermentation. However, in the present study HTL did not change glucose-stimulated insulin secretion and β-cell mass in diabetic rats, but HTL+CKJ additively improved insulinotropic action suggesting that HTL may have enhanced the action of chungkookjang. Thus, the anti-diabetic effects of HTL were related to enhanced insulin signaling decreasing hepatic gluconeogenesis, probably due to fructan in HTL. The different results may be due to the amount of oligofructan contents in the HTL diet. However, we cannot eliminate the possibility of stronger anti-diabetic effects with higher dosages of oligofructan from Jerusalem artichoke.

We previously demonstrated that chungkookjang exerts insulinotropic action, potentiating glucose-stimulated insulin secretion and increasing β-cell mass by elevating β-cell proliferation and decreasing apoptosis [6]. GLP-1 secreted from the intestinal L-cells increases insulin secretion from β-cells and expands β-cell mass after meals. GLP-1 or exendin-4, a GLP-1 receptor agonist, potentiates insulinotropic action by enhancing insulin/IGF-1 signaling in β-cells through increasing intracellular cAMP→ phosphorylation of cAMP responding element binding protein (CREB) →IRS-1 protein expression [32,33]. Genistein enhances insulinotropic action in β-cells by activating cAMP→CREB phosphorylation→ tyrosine phosphorylation of IRS2 [34,35]. In addition, isoflavnoids support glucose management through estrogen-like actions. Estrogen is reported to improve glucose homeostasis by potentiating glucose-stimulated insulin secretion and attenuates insulin resistance [16].

Soy protein also limits the accretion of visceral adiposity and increased peroxisome proliferator-activated receptor (PPAR)-γ and GLUT4 expressions, improving glucose metabolism in Wistar rats fed sucrose-rich diets [36]. Soy protein including isoflavonoids partially prevents insulin resistance, steatosis, and hypercholesterolemia in rats fed Western diets by reducing the expression of enzymes related to fatty acid synthesis such as nuclear sterol receptor element binding protein-1c and by increasing the proteins associated with fatty acid degradation such as PPAR-α [35]. However, Gobert et al. [37] reported that soy protein isolates do not significantly affect fasting or postprandial glucose or insulin, fasting HbA_1C_, or indices of insulin sensitivity. Thus, it remains unclear if soy protein has anti-diabetic effects. Our previous *in vitro* study demonstrated that water extracts of chungkookjang, containing mostly peptides, greatly increase insulin-stimulated glucose uptake compared to soybeans by increasing PPAR-γ expression [38]. The increase by water extracts is much greater than the methanol fraction of chungkookjang containing isoflavonoid [38], suggesting that peptides in chungkookjang are better for improving insulin resistance than those in soybeans.

## Conclusions

CKJ and HTL both had potent, but different, anti-diabetic effects in diabetic rats: CKJ potentiated glucose-stimulated insulin secretion and increased β-cell mass best, whereas HTL improved insulin sensitivity better than CKJ. CKJ+HTL exhibited partial additive and complementary anti-diabetic effects by potentiating insulinotropic action and reducing insulin resistance in diabetic rats.

## Abbreviation

HTL: Jerusalem artichoke (*Helianthus tuberosus Linne*); CKJ: Chungkookjang (fermented soybeans); Px: Pancreatectomy; AUC: Area under the curve; IRS: Insulin receptor substrate; PEPCK: Phosphoenolpyruvate carboxykinase; GLUT: Glucose transporter; ACC: Acetyl CoA carboxylase; GLP-1: Glucagon-like peptides; CREB: CAMP responding element binding protein; PPAR: Peroxisome proliferator-activated receptor.

## Competing interests

The authors declare that they no competing interests.

## Authors' contributions

YHJ and MJK performed isoflavonoid and amino acids analysis and statistical analysis of all data. SAK and DSK conducted animal study. DYK and SP conceived the experiment and wrote the manuscript. All authors read and approved the final manuscript.
